# Inhibition of Mg^2+^ Extrusion Attenuates Glutamate Excitotoxicity in Cultured Rat Hippocampal Neurons

**DOI:** 10.3390/nu12092768

**Published:** 2020-09-10

**Authors:** Yutaka Shindo, Ryu Yamanaka, Kohji Hotta, Kotaro Oka

**Affiliations:** 1 Department of Bioscience and Informatics, Faculty of Science and Technology, Keio University, Yokohama, Kanagawa 223-8522, Japan; shindo@z5.keio.jp (Y.S.); 1024mbmail@gmail.com (R.Y.); khotta@bio.keio.ac.jp (K.H.); 2 Faculty of Pharmaceutical Sciences, Sanyo-Onoda City University, Sanyo-Onoda, Yamaguchi 756-0884, Japan; 3 Waseda Research Institute for Science and Engineering, Waseda University, Shinjuku, Tokyo 162-8480, Japan; 4 Graduate Institute of Medicine, College of Medicine, Kaohsiung Medical University, Kaohsiung 80708, Taiwan

**Keywords:** magnesium, excitotoxicity, fluorescence imaging, neuroprotection

## Abstract

Magnesium plays important roles in the nervous system. An increase in the Mg^2+^ concentration in cerebrospinal fluid enhances neural functions, while Mg^2+^ deficiency is implicated in neuronal diseases in the central nervous system. We have previously demonstrated that high concentrations of glutamate induce excitotoxicity and elicit a transient increase in the intracellular concentration of Mg^2+^ due to the release of Mg^2+^ from mitochondria, followed by a decrease to below steady-state levels. Since Mg^2+^ deficiency is involved in neuronal diseases, this decrease presumably affects neuronal survival under excitotoxic conditions. However, the mechanism of the Mg^2+^ decrease and its effect on the excitotoxicity process have not been elucidated. In this study, we demonstrated that inhibitors of Mg^2+^ extrusion, quinidine and amiloride, attenuated glutamate excitotoxicity in cultured rat hippocampal neurons. A toxic concentration of glutamate induced both Mg^2+^ release from mitochondria and Mg^2+^ extrusion from cytosol, and both quinidine and amiloride suppressed only the extrusion. This resulted in the maintenance of a higher Mg^2+^ concentration in the cytosol than under steady-state conditions during the ten-minute exposure to glutamate. These inhibitors also attenuated the glutamate-induced depression of cellular energy metabolism. Our data indicate the importance of Mg^2+^ regulation in neuronal survival under excitotoxicity.

## 1. Introduction

Magnesium plays important roles in the nervous system [1,2]. Elevation of the Mg^2+^ concentration in cerebrospinal fluid (CSF) increases synapse formation, enhances recognition and learning abilities in rats [3], and causes neural stem cell proliferation in mice [4]. Further, a deficiency of Mg^2+^ in the brain is implicated in neuronal diseases, and some researchers have reported lower Mg^2+^ concentrations than normal in the brain of patients with neurodegenerative diseases [5,6]. In rats, significant loss of dopaminergic neurons in the substantia nigra, similar to the loss seen in Parkinson’s disease, was elicited merely by feeding them a low-magnesium diet for two generations [7]. In contrast, Mg^2+^ supplementation or overexpression of the Mg^2+^ channel have neuro-protective effects on cellular and animal models of Parkinson’s [8,9,10] and Alzheimer’s diseases [11]. These evidences show that Mg^2+^ is intricately involved in nervous system functioning and neuroprotection.

At the cellular level, Mg^2+^ is essential for maintaining enzymatic activities and energy metabolism, and its intracellular concentration is regulated by a number of ion channels and transporters on cell and organelle membranes [12,13,14,15,16]. Recent researches have revealed that Mg^2+^ also has more active roles in regulating intracellular signal transduction [17], cellular metabolism [18,19], and cell division [20]. These studies indicate that the concentration changes of Mg^2+^ is in a small range from 0.5 mM to 1.2 mM but essential for cellular functions. In neurons, the most widely known role of Mg^2+^ is the extracellular blockade and regulation of N-methyl-D-aspartate (NMDA) receptors, which occurs under physiologically normal extracellular concentrations [21,22]. In addition, intracellular Mg^2+^ is thought to play an indispensable role in neuronal functions [1]. Previously, we demonstrated that some neurotransmitters, as well as neuronal excitation itself, elicit changes in the intracellular Mg^2+^ concentration ([Mg^2+^]_i_) in cultured neurons [23,24,25,26]. In particular, glutamate induced interesting changes in [Mg^2+^]_i_. Although it is the most abundant neurotransmitter in the mammalian brain, excessive accumulation of glutamate around neurons induces neuronal cell death in a phenomenon known as excitotoxicity [27]. Toxic concentration of extracellular glutamate induced the release of Mg^2+^ from mitochondria, which is triggered by excessive Ca^2+^ accumulation in the mitochondria, leading to the increase in [Mg^2+^]_i_ [26]. Subsequently, the [Mg^2+^]_i_ turns to decrease. Given that Mg^2+^ deficiency is involved in neuronal diseases, the decreasing [Mg^2+^]_i_ phase likely has detrimental effects on neuronal survival, and thus plays a key role in excitotoxicity. However, the mechanism causing the decreasing [Mg^2+^]_i_ phase and its effect on excitotoxicity has not yet been elucidated.

In this study, we examined whether changes in [Mg^2+^]_i_ are involved in neuronal cell death via excitotoxicity in rat hippocampal neurons. We investigated the mechanisms involved in the phase in which the transient [Mg^2+^]_i_ increase in response to glutamate is reversed, and the effects of [Mg^2+^]_i_ on cellular energy metabolism. Quinidine and amiloride were used to inhibit cellular Mg^2+^ extrusion. Because those inhibitors are not specific for Mg^2+^ transport, it is important to confirm that both inhibitors show similar effects and that the effects were mediated via the changes in [Mg^2+^]_i_. Glutamate excitotoxicity results from continuous activation of the NMDA receptors on neurons, which leads to excessive Ca^2+^ influx into the cytosol and overload in the mitochondria, resulting in depolarization of the mitochondrial membrane potential and the release of cell death signals [27,28]. Mitochondria begin to release apoptosis signals within 10–20 min in response to toxic concentrations of glutamate [27]. Therefore, the Mg^2+^ transient elicited during the first 10 min of exposure to the glutamate stimulus may affect intracellular signals prior to the release of cell death signals under excitotoxicity. We investigated the hypothesis that the intracellular Mg^2+^ homeostasis and the intracellular Mg^2+^ regulatory system are key to cell protection in neuronal pathology.

## 2. Materials and Methods

### 2.1. Ethical Approval

All animal procedures were approved by the ethics committee of Keio University (permit number 09106-(7)). All methods were carried out in accordance with the relevant guidelines and regulations.

### 2.2. Dissociation Culture of Rat Hippocampal Neurons

The primary cultures of hippocampal neurons were prepared from day 18 embryonic Wistar rats (Charles River Laboratories Japan, Tokyo, Japan). Extracted hippocampi were dissociated using a dissociation kit (Sumitomo Bakelite, Tokyo, Japan). Isolated hippocampal neurons were plated on glass bottom dishes (Iwaki, Tokyo, Japan) coated with poly-D-lysine (PDL; Sigma-Aldrich, St. Louis, MO, USA) for fluorescence imaging or in 96-well plates for MTT assay, and cultured in neurobasal medium (Thermo Fisher Scientific, Waltham, MA, USA) supplemented with B-27 (Thermo Fisher Scientific, Waltham, MA, USA), 2 mM L-glutamine, 50 U/mL penicillin, and 50 μg/mL streptomycin (Nacalai Tesuque, Kyoto, Japan). The neurons were cultured at 37 °C in a humidified atmosphere of 5% CO_2_ for 7–9 days. Neuronal culture and synaptic formation among neurons were confirmed by immunofluorescence imaging of a neuron marker, βIII-tubulin (Sigma-Aldrich, St. Louis, MO, USA), and a synapse marker, synapsin I (Abcam, Cambridge, UK) (Figure S1).

### 2.3. MTT Assay

The MTT assay was conducted to measure cell viability. Neurons at the concentration of 8 × 10^3^ cells/well were cultured on 96-well plates for 7 days. Neurons cultured in 96-well plates were incubated in culture medium with or without an inhibitor of Mg^2+^ extrusion (quinidine at 200 μM or amiloride at 500 μM; Sigma-Aldrich) and/or an inhibitor of mechanistic target of rapamycin (mTOR) (Torin1 at 2.5 μM; Chem Scene, Monmouth Junction, NJ, USA) for 10 min. Next, 100 μM glutamate with 10 μM glycine or control culture medium was applied and cells were incubated for 10 min. The culture medium containing inhibitor and/or glutamate was then replaced with normal culture medium, and the neurons were incubated for 24 h. The neurons were then incubated in culture medium containing 0.5 mg/mL MTT (Nacalai Tesuque). The culture medium was removed and 100 μL of dimethyl sulfoxide (DMSO; Nacalai Tesuque) was added to each well to dissolve the precipitate, and absorbance at 570 nm was measured using a microplate reader, Fluoroskan Ascent FL (Thermo Fisher Scientific). Cell viability in the treatment cultures is expressed as a proportion of cell viability in the control cultures.

### 2.4. Simultaneous Fluorescence Imaging of Intracellular Mg^2+^ and Ca^2+^

Changes in [Mg^2+^]_i_ and intracellular Ca^2+^ concentration ([Ca^2+^]_i_) were measured by simultaneous imaging using the probes KMG-104-AM [29] and Fura-Red-AM (Thermo Fisher Scientific), respectively. KMG-104 is sufficiently selective to measure the Mg^2+^ signal without interference from the Ca^2+^ signal [29,30]. Neurons cultured on glass-bottom dishes were washed with Hanks’ balanced salt solution (HBSS; NaCl 137 mM, KCl 5.4 mM, CaCl_2_ 1.3 mM, MgCl_2_ 0.5 mM, MgSO_4_ 0.4 mM, Na_2_HPO_4_ 0.3 mM, KH_2_PO_4_ 0.4 mM, NaHCO_3_ 4.2 mM, D-glucose 5.6 mM, HEPES 10 mM, pH adjusted to 7.4 with NaOH) and incubated in HBSS containing 5 μM KMG-104-AM, 10 μM Fura-Red-AM and 0.02% pluronic F-127 (Thermo Fisher Scientific) at 37 °C for 30 min. The neurons were then washed twice and incubated in HBSS, in HBSS without CaCl_2_ (nominally Ca^2+^-free HBSS) for measurements under Ca^2+^-free conditions, for 15 min at 37 °C to allow for complete hydrolysis of the acetoxymethyl (AM) ester.

Fluorescence measurements were performed using fluorescence microscope, ECLIPSE TE300 (Nikon, Tokyo, Japan) equipped with 10× and 20× objective lenses, S Fluor (Nikon). Excitation light with a wavelength of approximately 488 nm was selected from a 150W Xe lamp using a monochrometer unit (Hamamatsu photonics, Shizuoka, Japan). The fluorescence signals that passed through a 510 nm dichroic mirror were separated using a 590 nm dichroic mirror and detected with a CCD camera, HiSCA (Hamamatsu photonics) using a 535/55 nm band pass filter for KMG-104 and a 600 nm long-pass filter for Fura-Red, respectively. Time-lapse images were acquired every 5 sec. Fluorescence intensity (F) was calculated as the mean intensity in a region of interest (ROI) containing the entire cell body by using Aquacosmos software (Hamamatsu photonics). The values of F recorded during time-lapse imaging were normalized by the initial fluorescence intensity (F_0_) of each cell. The KMG-104 data were presented and analyzed as F/F_0_. Since a decrease in the fluorescence intensity of Fura-Red indicates an increase in [Ca^2+^], the Fura-Red data were presented and analyzed as F_0_/F.

### 2.5. Fluorescence Imaging of Mitochondrial Membrane Potential

Mitochondrial membrane potential was measured using tetramethylrhodamine ethyl ester (TMRE; Thermo Fisher Scientific). The neurons were washed with HBSS and then incubated for 20 min in HBSS containing 25 nM TMRE. Next, the dye was diluted to 2.5 nM in HBSS, and the neurons were incubated for an additional 10 min to equilibrate the dye. For measurements under high Mg^2+^ conditions, cells were stained and further incubated in HBSS with 8 mM Mg^2+^.

Fluorescence of the TMRE was measured using the fluorescence microscope, ECLIPSE TE300. Excitation light with a wavelength of approximately 560 nm was selected from a 150W Xe lamp using the monochrometer unit. The fluorescence signals that passed through a 580 nm dichroic mirror and a 600 nm long-pass filter were detected with the CCD camera. F was calculated as the mean fluorescence intensity in a ROI containing the entire cell body. The values of F recorded during time-lapse imaging were normalized by the F_0_ of each cell, and the data were presented and analyzed as F/F_0_.

### 2.6. Fluorescence Imaging of Intracellular ATP

To compare intracellular ATP concentrations, genetically encoded ATP sensor, ATeam [31], was induced in neurons using an adeno-associated virus (AAV) vector. 30 μL of the AAV vector were added to every dish and neurons were incubated for 3–7 days to ensure sufficient expression of the sensor protein. After washing the cells twice with HBSS, or with HBSS with 8 mM Mg^2+^ for measurements under high Mg^2+^ conditions, the fluorescence was measured.

Fluorescence imaging of the neurons was performed using a confocal laser scanning microscope system, FV-1000 (Olympus, Tokyo, Japan) with a 40× oil-immersion objective lens. ATeam was excited at 440 nm from a diode laser. The fluorescence signals were separated using a 510 nm dichroic mirror and observed at 460–500 nm for cyan fluorescent protein (CFP) and 515–615 nm for yellow fluorescent protein (YFP). Fluorescence intensity was calculated as the mean intensity in a ROI containing the entire cell body. The intracellular ATP levels are represented by the YFP to CFP fluorescence ratio (ATeam ratio).

### 2.7. Statistical Analysis

For multiple testing, analysis of variance (ANOVA) was performed, and then Dunnett’s test was used to compare the control group with the other groups or Tukey’s test was used to compare all possible combinations of groups, assuming parametric distribution. *p* < 0.05 was considered significantly different. 

## 3. Results

### 3.1. Inhibitors of Mg^2+^ Extrusion Attenuated the Excitotoxicity

**Figure 1:**
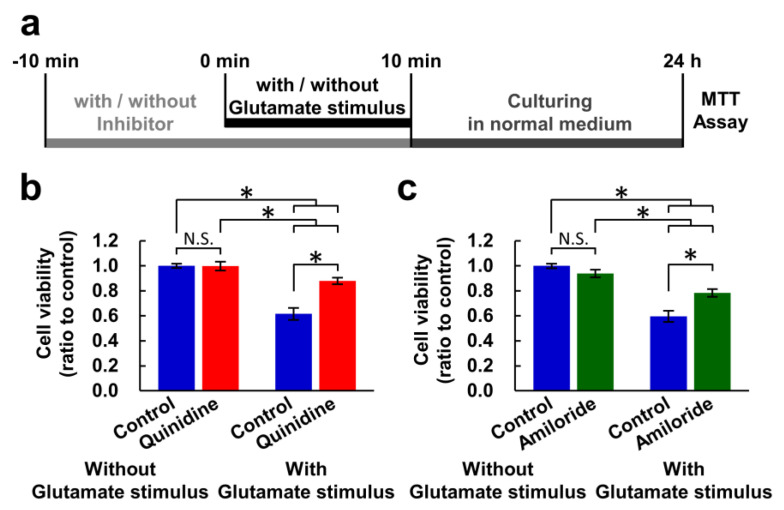
Inhibitors of Mg^2+^ extrusion attenuated excitotoxicity. (**a**) Experimental procedure of the MTT assay for measuring the effect of a ten-minute glutamate stimulus. Comparison of cell viability in the presence or absence of quinidine (200 μM) (**b**) or amiloride (500 μM) (**c**) (*n* = 30 for each). Error bars indicate SEM. * indicates *p* < 0.05 among all possible combinations by Tukey’s test. N.S. indicates no statistically significant difference.

Previous studies have indicated that cells express several Mg^2+^ extrusion mechanisms, and quinidine, amiloride, and imipramine are used to inhibit these Mg^2+^ extrusion mechanism [32,33]. Because these inhibitors are not specific for Mg^2+^ transport, we checked whether both quinidine and amiloride have similar effect on neurons. First, we examined whether inhibitors of Mg^2+^ extrusion mechanisms had an effect on cell viability under excitotoxic conditions. A combination of 100 μM glutamate and 10 μM glycine was used as an excitotoxic glutamate stimulus. It has been demonstrated that even 10 min of exposure to toxic concentrations of glutamate induces, to some extent, neuronal cell death 24 h after the stimulation [27]. Here, we aimed to assess the impact of the glutamate stimulus for 10 min on cell viability 24 h after the stimulus and to evaluate the effects of inhibition of Mg^2+^ extrusion on it. We therefore replaced the culture medium for a fresh one not containing glutamate or inhibitors after ten-minute exposure to glutamate stimulus in the presence or absence of an inhibitor to assess the effects of the glutamate stimulus and the inhibition of Mg^2+^ extrusion only during the ten-minute glutamate stimulus. Then, the cell viability after 24 h was measured with MTT assay (Figure 1a). The ten-minute exposure to glutamate stimulus induced cell death in approximately 40% of the neurons after 24 h (controls with glutamate stimulus in Figure 1b,c). This experimental condition, which induces 40% cell death, was adopted in this study because it is easy to assess the effect of inhibitors on cell viability, whether to accelerate or suppress the toxicity. Both inhibitors partially attenuated this decrease in cell viability, while exposure to the inhibitors for 20 min without glutamate stimulus had no effect on viability (Figure 1b,c). 

### 3.2. The Inhibitors Suppressed the Decreasing Phase of the Glutamate-Induced Mg^2+^ Transient

**Figure 2:**
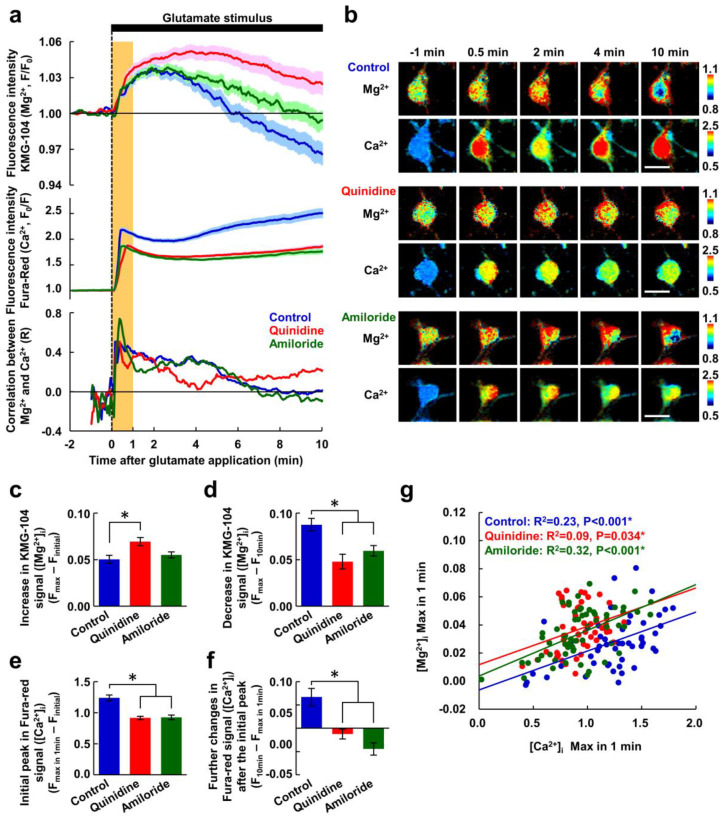
Inhibitors of Mg^2+^ extrusion suppressed the decreasing phase of the glutamate-induced Mg^2+^ transient. (**a**) Time-courses of [Mg^2+^]_i_ (upper) and [Ca^2+^]_i_ (middle) responses to the glutamate stimulus, and the correlation between them (bottom) in the presence or absence of inhibitor (blue: control, *n* = 52 cells; red: with quinidine at 200 μM, *n* = 48 cells; green: with amiloride at 500 μM, *n* = 64 cells; from 5 replicates each). Mg^2+^ and Ca^2+^ were simultaneously measured in the same neurons. Data are presented as the average (solid line) ± SEM (shaded area). The glutamate stimulus (100 μM glutamate with 10 μM glycine) was applied at 0 min (dotted line). The period 0–1 min is shaded orange. (**b**) Pseudo-colored images showing representative responses of Mg^2+^ and Ca^2+^ in each condition. Scale bars, 20 μm. Comparisons of amplitudes of increase in KMG-104 signal ([Mg^2+^]_i_) (**c**), decrease in KMG-104 signal ([Mg^2+^]_i_) (**d**), initial peak in Fura-red signal ([Ca^2+^]_i_) (**e**), and further changes in Fure-red signal ([Ca^2+^]_i_) after the initial peak (**f**) among the conditions extracted from the data shown in (**a**). Error bars indicate SEM. * indicates *p* < 0.05 compared with control by Dunnett’s test. (**g**) Scatter plot of maximum values of [Mg^2+^]_i_ and [Ca^2+^]_i_ within the first minute (orange area in Figure 2a) and their regression lines (blue: control; red: with quinidine; green: with amiloride). *p* values were determined from the correlation coefficient and the number of plots.

To confirm the effects of the inhibitors on cellular ion transports immediately after the application of glutamate stimulus, [Mg^2+^]_i_ and [Ca^2+^]_i_ were simultaneously visualized using KMG-104 and Fura-Red, respectively. The glutamate stimulus elicited a gradual increase in [Mg^2+^]_i_ and a steep increase in [Ca^2+^]_i_, after which [Mg^2+^]_i_ began to decrease. Within 10 min, [Mg^2+^]_i_ in the control was below the initial concentration (Figure 2a). Quinidine (200 μM) and amiloride (500 μM) partially suppressed both the [Mg^2+^]_i_ decrease and the [Ca^2+^]_i_ increase, but did not suppress the [Mg^2+^]_i_ increase (Figure 2a,b). Distributions of the increases in [Mg^2+^]_i_ and [Ca^2+^]_i_ were different (Figure 2b). In addition, no morphological changes such as cell body rounding or neurite fragmentation were observed, indicating that the neurons are still alive at 10 min. Quinidine enhanced the [Mg^2+^]_i_ increase (Figure 2c), and both quinidine and amiloride suppressed the Mg^2+^ decrease (Figure 2d). This indicates that glutamate stimulus simultaneously induced both Mg^2+^ mobilization and Mg^2+^ extrusion, which restricts amplitude of the increase in [Mg^2+^]_i_. As a result, neurons maintained a higher [Mg^2+^]_i_ level in the presence of the inhibitors than that in the control during the ten-minute exposure to glutamate stimulus. The inhibitors also partially attenuated the [Ca^2+^]_i_ peak observed within 1 min of the addition of the glutamate stimulus (Figure 2e). Following the initial peak, while the neurons in the control exhibited a further gradual increase in [Ca^2+^]_i_ to a level higher than the initial peak, in the presence of the inhibitors, [Ca^2+^]_i_ showed plateau after 4 min and did not exceed the initial peak level (Figure 2a middle panel,b,f). This was probably due to suppression of the Ca^2+^ influx via the voltage-gated Ca^2+^ channels (VGCC) by the higher [Mg^2+^]_i_ [34].

Under all three conditions, the changes in [Mg^2+^]_i_ and [Ca^2+^]_i_ were well correlated with each other immediately after the application of glutamate stimulus, and the correlation coefficient decreased with time (Figure 2a bottom panel). To further analyze the relationship between the [Ca^2+^]_i_ increase and the increasing phase of [Mg^2+^]_i_, we examined the correlation of the [Mg^2+^]_i_ and [Ca^2+^]_i_ maxima observed within 1 min of glutamate stimulus (Figure 2g). These correlated well in control neurons, because the increase in [Mg^2+^]_i_ is triggered by the increase in [Ca^2+^]_i_ as previously shown [26]. In the presence of the inhibitors, these maxima were still correlated, and although their regression lines shifted upward, they were almost parallel with each other (Figure 2g). This indicates that the inhibitors attenuated the [Ca^2+^]_i_ response, without affecting the Mg^2+^ response, immediately after the stimulus.

### 3.3. Neurons Extruded Mg^2+^ in Response to the Glutamate Stimulus

**Figure 3:**
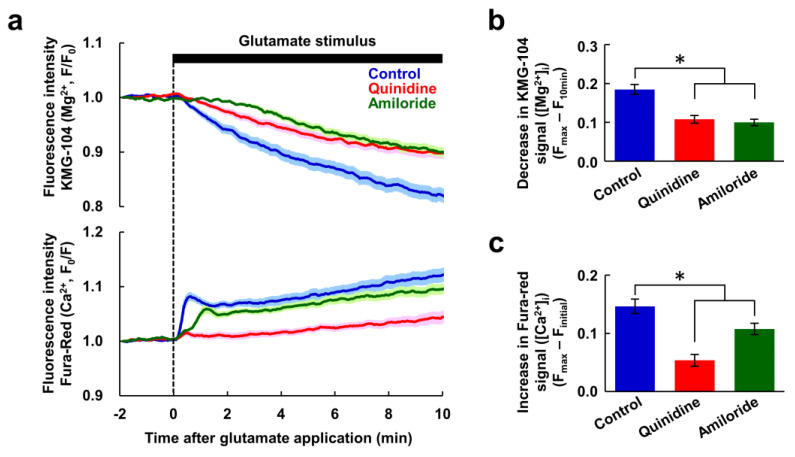
The Mg^2+^ and Ca^2+^ increases were not required for Mg^2+^ extrusion. (**a**) Time-courses of [Mg^2+^]_i_ (upper) and [Ca^2+^]_i_ (bottom) response to glutamate stimulus in nominally Ca^2+^-free conditions in the presence or absence of inhibitor (blue: control, *n* = 60 cells; red: with quinidine at 200 μM, *n* = 52 cells; green: with amiloride at 500 μM, *n* = 54 cells, from 5 replicates each). Data are presented as the average (solid line) ± SEM (shaded area). Comparison of decrease in KMG-104 signal ([Mg^2+^]_i_) (**b**) and increase in Fura-red signal ([Ca^2+^]_i_) (**c**) at 10 min after application of the glutamate stimulus extracted from the data shown in (**a**). Error bars indicate SEM. * indicates *p* < 0.05 compared with control by Dunnett’s test.

Next, we investigated whether the Ca^2+^ signal is a prerequisite for the Mg^2+^ extrusion. To do this, glutamate-induced responses were observed in nominally Ca^2+^-free conditions. Under this conditions, there was only a small [Ca^2+^]_i_ response, and the [Mg^2+^]_i_ increase was completely abolished. However the decrease in [Mg^2+^]_i_ remained (Figure 3a). Combined with Figure 2g, this suggests that the [Mg^2+^]_i_ increasing phase is Ca^2+^-dependent event. Both quinidine and amiloride suppressed this Mg^2+^ decrease to a similar extent (Figure 3b). Quinidine abolished the small [Ca^2+^]_i_ increase evoked by the glutamate stimulus, and amiloride partially attenuated it (Figure 3c). This suggests that both inhibitors affect the glutamate-induced Ca^2+^ mobilization other than Ca^2+^ influx from extracellular medium. This might have some contribution to the attenuation of glutamate-induced Ca^2+^ response by these inhibitors shown in Figure 2. These results also indicate that the decrease in [Mg^2+^]_i_ does not require the preceding [Mg^2+^]_i_ and [Ca^2+^]_i_ increases. This means that the [Mg^2+^]_i_-decreasing phase is not a homeostatic cellular response attempting to maintain a normal [Mg^2+^]_i_, but rather a glutamate-induced activation of Mg^2+^ extrusion via channels and/or transporters, and that this occurs in response to a neuronal excitation or an intracellular signal other than Ca^2+^. Taken together, these results indicate that glutamate stimulus induces both Ca^2+^-dependent Mg^2+^ release from the mitochondria and Mg^2+^ extrusion from the cytosol, simultaneously.

### 3.4. Effect of the Inhibition of the Mg^2+^ Extrusion on Cellular Energy Metabolism

**Figure 4:**
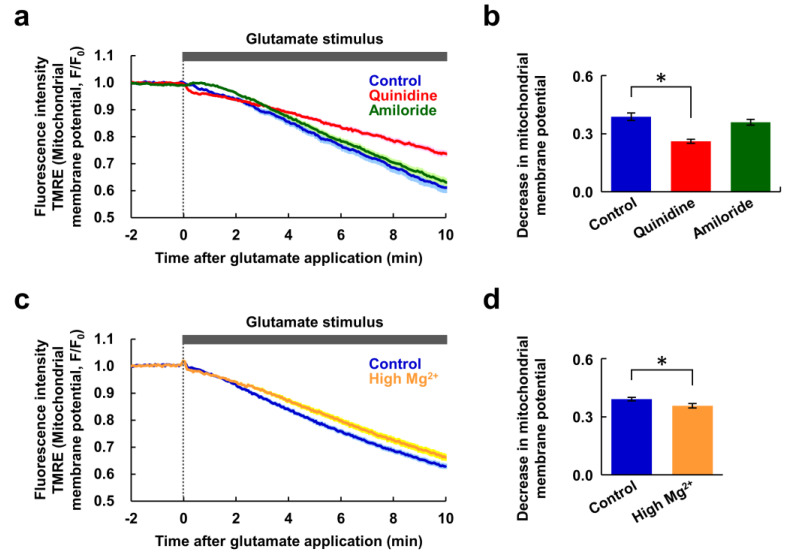
Changes in mitochondrial membrane potential in response to glutamate stimulus. (**a**) Time-courses of changes in mitochondrial membrane potential in response to the glutamate stimulus (blue: control, *n* = 90 cells; red: with quinidine at 200 μM, *n* = 89 cells; green: with amiloride at 500 μM, *n* = 100 cells, from 5 replicates each). Data are presented as the average (solid line) ± SEM (shaded area). (**b**) Comparison of decreases in mitochondrial membrane potential after 10 min extracted from the data shown in (**a**). Error bars indicate SEM. * indicates *p* < 0.05 compared with control by Dunnett’s test. (**c**) Time-courses of changes in mitochondrial membrane potential in response to the glutamate stimulus (blue: control, *n* = 75 cells; yellow: under high Mg^2+^ (8 mM) condition, *n* = 80 cells, from 5 replicates each). Data are presented as the average (solid line) ± SEM (shaded area). (**d**) Comparison of decreases in mitochondrial membrane potential after 10 min extracted from the data shown in (**c**). Error bars indicate SEM. * indicates *p* < 0.05 by t-test.

Next, we investigated whether inhibitors of Mg^2+^ extrusion had any effect on cellular energy metabolism. Since mitochondria play a central role in neuronal ATP synthesis, changes in mitochondrial membrane potential during glutamate stimulus were measured. In response to the glutamate stimulus, mitochondrial membrane potential decreased gradually (Figure 4a). It was still decreasing at 10 min, indicating the neurons still alive at this point. Quinidine partially attenuated this decrease, but amiloride had no effect on it (Figure 4b). Thus, although these inhibitors attenuated the glutamate-induced [Ca^2+^]_i_ increase to the same extent (Figure 2), amiloride did not suppress the depolarization of mitochondrial membrane potential. To further confirm the contribution of Mg^2+^ for maintenance of mitochondrial membrane potential, glutamate stimulus-induced changes in the mitochondrial membrane potential under high Mg^2+^ conditions were compared to those under normal conditions. High Mg^2+^ partially attenuated the decrease in mitochondrial membrane potential (Figure 4c,d), presumably by keeping [Mg^2+^]_i_ high. This supports the idea that Mg^2+^ has a protective effect on depolarization of mitochondrial membrane potential.

**Figure 5:**
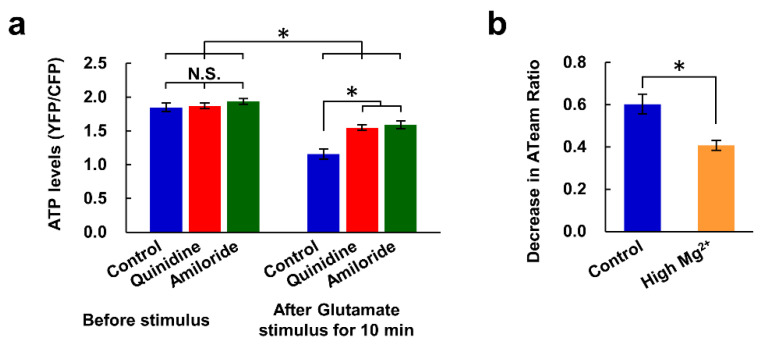
Inhibitors of Mg^2+^ extrusion attenuated the glutamate-induced decrease in intracellular ATP level. (**a**) Comparison of intracellular ATP levels (YFP/CFP ratio of ATeam) before and 10 min after application of the glutamate stimulus in the presence or absence of inhibitor (*n* = 10, 16, 18 cells from 6 replicates for each). Error bars indicate SEM. * indicates *p* < 0.05 among all possible combinations by Tukey’s test. N.S. indicates no statistically significant difference. (**b**) Comparison of decrease in ATeam ratio induced by glutamate stimulus in 10 min under control and high Mg^2+^ (8 mM) conditions (control: *n* = 32 cells from 9 replicates; High Mg^2+^: *n* = 34 cells from 11 replicates). Error bars indicate SEM. * indicates *p* < 0.05 by t-test.

We also investigated whether the inhibition of Mg^2+^ extrusion and the resulting high [Mg^2+^]_i_ lead to the maintenance of cellular ATP concentrations during the excitotoxicity process. Intracellular ATP concentrations were compared using a genetically encoded ATP sensor, ATeam [31]. The presence of quinidine or amiloride did not affect ATP levels in steady-state neurons, but both inhibitors partially attenuated the decrease in ATP levels evoked by the glutamate stimulus (Figure 5a). High Mg^2+^ also suppressed the glutamate-induced decrease in ATP level (Figure 5b). These results suggest that the high [Mg^2+^]_i_ resulting from the inhibition of Mg^2+^ extrusion contributes to the maintenance of intracellular ATP levels.

### 3.5. Involvement of mTOR in the Attenuation of Excitotoxicity by Mg^2+^

**Figure 6:**
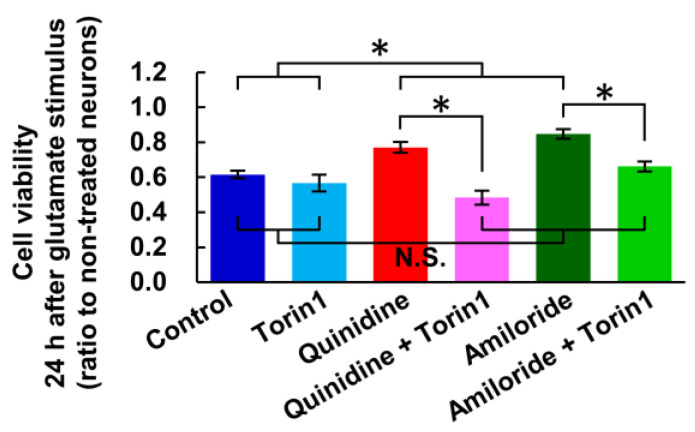
Inhibitor of mTOR abolished the attenuation of excitotoxicity by inhibiting Mg^2+^ extrusion. Comparison of cell viability 24 h after ten-minute glutamate stimulus with or without inhibitor of mTOR (Torin1: 2.5 μM) and/or inhibitors of Mg^2+^ extrusion (Quinidine: 200 μM, Amiloride: 500 μM) (*n* = 12, 9, 12, 12, 12, 12 for each). Error bars indicate SEM. * indicates *p* < 0.05 among all possible combinations by Tukey’s test. N.S. indicates no statistically significant difference.

In the previous study, we demonstrated that increase in [Mg^2+^]_i_ activates mTOR in cultured hippocampal neurons [23]. mTOR is an important signal implicated in the regulation of energy metabolism, cell growth and cell death [35,36]. We therefore examined the effect of mTOR inhibitor, Torin1, on the glutamate-induced neuronal cells death and its attenuation by the inhibitors of Mg^2+^ extrusion, according to the procedure shown in Figure 1a. While torin1 did not enhance the toxicity of glutamate stimulus, it abolished the attenuation of the toxicity by quinidine or amiloride (Figure 6). This suggests that the mTOR signal is involved in the attenuation of excitotoxicity by increasing [Mg^2+^]_i_.

## 4. Discussion

In this study, we demonstrated that inhibitors of Mg^2+^ extrusion attenuate glutamate excitotoxicity in cultured rat hippocampal neurons (Figure 1). A toxic concentration of glutamate induced Mg^2+^ extrusion from neurons, which was suppressed by both quinidine and amiloride (Figure 2 and Figure 3). These inhibitors also attenuated the glutamate-induced depression of cellular energy metabolism (Figure 5). The protective effect of those inhibitors against excitotoxicity was abolished by inhibition of mTOR (Figure 6). While quinidine and amiloride partially attenuated the glutamate-evoked [Ca^2+^]_i_ response (Figure 2e,f), in particular, mobilization of Ca^2+^ from other than extracellular medium (Figure 3), they did not suppress the increasing phase of the [Mg^2+^]_i_ transient (Figure 2c), in spite of its Ca^2+^-dependency (Figure 2g and Figure 3). Amiloride also had no effect on the glutamate-induced mitochondrial depolarization (Figure 4), despite attenuating the cytosolic Ca^2+^ increase (Figure 2e,f). Given that the depolarization of mitochondrial membrane potential is a critical event in the process of excitotoxicity [27], the partial attenuation of the [Ca^2+^]_i_ increase by the amiloride does not affect glutamate excitotoxicity. Therefore, the neuroprotective effect of the inhibitors is probably due to the maintenance of high [Mg^2+^]_i_ during the glutamate stimulus. This idea is also supported by the results that glutamate-induced decrease in mitochondrial membrane potential and cytosolic ATP level were partially attenuated under high Mg^2+^ conditions (Figure 4c,d and Figure 5b), presumably by keeping [Mg^2+^]_i_ high. Our data therefore indicate the importance of Mg^2+^ regulation in neuronal cell death and survival.

One of the key roles of Mg^2+^ in ensuring cell survival involves the regulation of mitochondrial functions [37]. It affects enzymatic activities in the tricarboxylic acid (TCA) cycle in mitochondria; thus, a decrease in mitochondrial Mg^2+^ concentration results in a downregulation of mitochondrial membrane potential and ATP synthesis [38,39,40]. On the other hand, extra-mitochondrial Mg^2+^ suppresses mitochondrial Ca^2+^ uptake [41] which depolarizes mitochondrial membrane potential. Either or both of these processes might contribute to the attenuation of mitochondrial membrane potential depolarization by quinidine (Figure 4) and the suppression of the decrease in ATP levels caused by quinidine and amiloride (Figure 5). The idea that elevated [Mg^2+^]_i_ contributes to the maintenance of cellular ATP levels was also supported by our previous work [42]. Although amiloride did not inhibit the depolarization of mitochondrial membrane potential (Figure 4), it did suppress the decrease in ATP levels and cell death (Figure 1 and Figure 5). This suggests that Mg^2+^ affects not only mitochondrial functions but also other mechanisms that are involved in maintaining the cellular ATP concentration and cell viability.

One of the ways in which Mg^2+^ might do this is by regulating intracellular signal transductions [17]. In particular, mTOR is considered an important downstream target of Mg^2+^ [18,19,43]. The mTOR pathway is implicated in the regulation of cellular metabolism and proliferation [36]. In pancreatic cancer cells, overexpression of SLC41A1, a membrane protein for Mg^2+^ extrusion [32], and the resulting low [Mg^2+^]_i_, inhibited the Akt/mTOR pathway and cancer proliferation [44]. In cultured hippocampal neurons, [Mg^2+^]_i_ within the physiological concentration range is correlated with mTOR activity [23]. These findings strongly suggest that Mg^2+^ is a key regulator of mTOR and mTOR-related signal transductions. Inhibition of the Akt/mTOR pathway in cultured neurons in response to toxic concentrations of glutamate has been reported [45]. This inhibition might result from the glutamate-induced decrease in [Mg^2+^]_i_ demonstrated in this study (Figure 2 and Figure 3). The fact that the Akt/mTOR signal is also involved in regulation of both cellular ATP synthesis and also apoptotic signals [35,46,47] would explain why maintaining a high [Mg^2+^]_i_ prevent neuronal cell death. In this study, we showed that inhibition of mTOR abolished the attenuation of glutamate excitotoxicity by inhibiting Mg^2+^ extrusion (Figure 6). This suggests that maintaining high [Mg^2+^]_i_ protect neurons against excitotoxicity via the mTOR signal.

**Figure 7:**
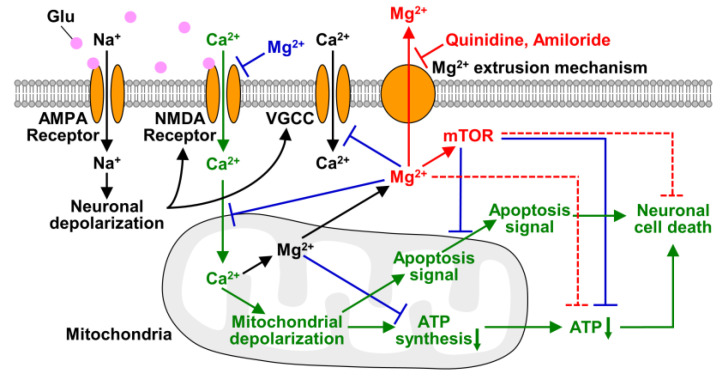
Schematic of roles of Mg^2+^ in excitotoxicity. The transport and effects of Mg^2+^ and mTOR demonstrated in this study are shown in red. In this study, we demonstrated that glutamate stimulus induces not only Mg^2+^ released from mitochondria but also Mg^2+^ extrusion from the cytosol, and quinidine and amiloride inhibit it. Maintaining Mg^2+^ in neurons suppressed glutamate-induced decrease in cellular ATP level and also attenuated neuronal cell death via mTOR signaling pathway. Dotted lines indicate pathways that are still obscure whether they are direct or indirect effects. The effects of Mg^2+^ demonstrated previous studies are shown in blue, and the main excitotoxic signals are shown in green.

As discussed above and summarized in Figure 7, Mg^2+^ is a key factor in saving neurons from neurodegenerative diseases. Since inhibition of cellular Mg^2+^ extrusion and resulting maintenance of high [Mg^2+^]_i_ protect cells from neurodegenerative disorders, the Mg^2+^ extrusion mechanisms might be a therapeutic target for neuronal diseases. Among Mg^2+^ channels and transporters, SLC41A1 is considered to be the major transporter for cellular Mg^2+^ extrusion [32,48], while it was also suggested that cells has several different mechanisms for extruding cytosolic Mg^2+^ [49]. Preventing cellular Mg^2+^ loss by inhibiting such transporters might delay the progression of neurodegeneration. Because Mg^2+^ is a broad-spectrum regulator of cellular metabolisms and signaling, change in [Mg^2+^]_i_ affects multiple aspects of the process of excitotoxicity, even though it is not a main signal for inducing neuronal cell death (Figure 7). In present study, we have demonstrated that keeping Mg^2+^ inside cells is important for neuronal survival under conditions of cellular stress. It may protect neurons from neurodegeneration also in vivo because it has been reported that gain-of-function mutation in SLC41A1, that leads to enhanced Mg^2+^ release from cytosol, and mutation in a Mg^2+^ and Ca^2+^ permeable channel, TRPM7, that attenuates cellular Mg^2+^ uptake, are associated with the pathogenesis of neurodegenerative diseases in human brain [50,51]. To support this, further studies on Mg^2+^ dynamics in vivo are needed. Our results demonstrated in this study suggest the potential of cellular Mg^2+^ homeostasis and the Mg^2+^ transport system for therapy and the prevention of neurodegenerative diseases.

## Supplementary Materials

The following are available online at https://www.mdpi.com/2072-6643/12/9/2768/s1, Figure S1: Immunofluorescence images of a synapse marker.
