# Supplementary material for: Inhibition of Mg2+ Extrusion Attenuates Glutamate Excitotoxicity in Cultured Rat Hippocampal Neurons
Source: Nutrients. 2020 Sep 10;12(9):2768. doi: 10.3390/nu12092768 (PMC7551965; doi:10.3390/nu12092768)
Supplement: Supplementary file 1 [file nutrients-12-02768-s001.pdf]

Supporting Information

Inhibition of Mg^{2+} extrusion attenuates glutamate excitotoxicity in cultured rat hippocampal neurons

Yutaka Shindo[1], Ryu Yamanaka[1,2], Kohji Hotta[1], Kotaro Oka[1,3,4]

1: Department of Bioscience and Informatics, Faculty of Science and Technology, Keio University, Yokohama, Kanagawa, Japan

2: Faculty of Pharmaceutical Sciences, Sanyo-Onoda City University, Sanyo-Onoda, Yamaguchi, Japan.

3: Waseda Research Institute for Science and Engineering, Waseda University, Shinjuku, Tokyo, Japan

4: Graduate Institute of Medicine, College of Medicine, Kaohsiung Medical University, Kaohsiung, Taiwan

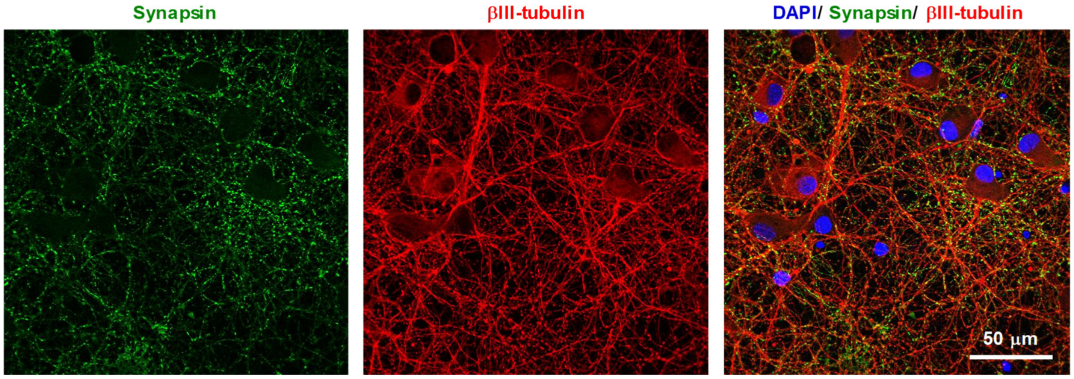

Figure S1. Immunofluorescence images of a synapse marker, synapsin I (green), and a neuron marker, βIII-tubulin (red), and merged image (blue: nucleus stained by DAPI, green synapsin I, Red: βIII-tubulin), in rat hippocampal neurons cultured for 7 days.

Immunofluorescence imaging

Cultured neurons were fixed in 4% paraformaldehyde (PFA; Nacalai Tesque, Kyoto, Japan) diluted in PBS for 20 min at room temperature. Samples were washed twice in PBS to remove residual PFA. They were then permeabilized in PBS with 0.1% Triton X-100 (Nacalai Tesque) (PBT) for 1 min and incubated in PBT with 10% goat serum for 30 min for blocking. Mouse anti-βIII-tubulin (1:250, Sigma-Aldrich, St. Louis, MO, USA) and rabbit anti-synapsin I (1:1000, Abcam, Cambridge, UK) were diluted in blocking solution and applied to the samples for overnight at 4°C. The samples were then washed three times with PBT and incubated in blocking solution containing 4′,6-diamidino-2-phenylindole (DAPI; 1:1000, Dojindo, Tokyo, Japan), Alexa Fluor 488 anti-rabbit IgG, and Alexa Fluor 633 anti-mouse IgG (each at 1:1000, Thermo Fisher Scientific, Waltham, MA, USA) for 40 min at room temperature. The samples were washed three times with PBT and then filled with PBS.

The samples were observed using a confocal laser scanning microscope system (FluoViewFV1000, Olympus, Tokyo, Japan) mounted on an inverted microscope with ×60 oil immersion objectives. DAPI, Alexa Fluor 488, and Alexa Fluor 633 were sequentially excited with lasers at 405 nm, 488 nm and 635 nm, respectively, and the fluorescence was observed at 425–475 nm for DAPI, 500–545 nm for Alexa Fluor 488 and 655–755 nm for Alexa Fluor 633.
